# Acute confusional state caused by Hashimoto's encephalopathy in a patient with hypothyroidism: a case report

**DOI:** 10.4076/1757-1626-2-7967

**Published:** 2009-06-19

**Authors:** Pramil Cheriyath, Vinod Nookala, Anupam Srivastava, Salim Qazizadeh, Daniel Fischman

**Affiliations:** 1 Department of Internal Medicine205 S Front Street, BMAB 3-C, Harrisburg Hospital, PinnacleHealth Systems, Harrisburg, PA 17104USA; 2 Department of Endocrinology, Harrisburg HospitalHarrisburg, PA 17104USA; 3 Department of Neurology, Harrisburg HospitalHarrisburg, PA 17104USA

## Abstract

**Introduction:**

Hashimoto's Encephalopathy is an unusual condition associated with Hashimoto's Thyroiditis. This immune-mediated, steroid-dependent entity was first described thirty years ago. In this case report, we discuss the importance of considering this diagnosis in the evaluation of confusion.

**Case presentation:**

The patient is a 55-year-old African-American woman residing in United States, who was admitted to the hospital with a four-day history of mental status changes. Her past medical history was significant for type II Diabetes Mellitus, Hypertension, and Hypothyroidism. There was no reported seizure activity. The patient's vital signs were stable on admission. On examination, the patient was awake, alert, oriented to place and time. Her neurological examination revealed agraphia and dyslexia. Her speech showed lack of fluency and hesitation. Her complete blood count and electrolytes were within normal limits. The patient's brain CT scan did not reveal any significant findings. Her Magnetic Resonance Imaging only revealed mild chronic microangiopathy, which caused by “small vessel disease.” Her Electroencephalogram did not reveal any finding consistent with seizure activity. Cerebral spinal fluid analysis was likewise did not reveal a cause for this patient's acute onset of confusion. In contrast to the above negative finding, this patient's Thyroid stimulating hormone was discovered to be 15 UIU/ml. She was subsequently given 1000 mg of intravenous Methylprednisolone daily for 3 days. This three-day course of high-dose, intravenous steroids resulted complete resolution of the patient's symptoms. She was then discharged on an eleven-day course of oral prednisone 60 mg.

**Conclusion:**

Hashimoto's Encephalopathy should be considered in the differential diagnosis of an acute confusional state since it is responsive to steroid therapy and represents a readily reversible cause acute mental status changes. Clues to this diagnosis include elevated antithyroid antibodies, abnormal Thyroid stimulating hormone values, and exclusion of other causes of acute mental status changes.

## Introduction

Hashimoto's encephalopathy (HE) is a rare clinical syndrome that has been associated with elevated anti thyroid antibodies. As a result of confusion regarding any association with hypothyroidism, HE is now more frequently known by the name “Steroid Responsive Autoimmune Thyroiditis (SREAT)”.

This immune-mediated, steroid-dependent entity was first described in 1966 [1]. It is more commonly seen in women. Since this condition can result in seizure activity and requires treatment beyond simple hormone replacement, it must be entertained in the differential diagnosis of mental status changes in a patient with hypothyroidism. We present the case of one such patient who presented with an acute confusional state due to Hashimoto's encephalopathy.

## Case presentation

A 55-year-old African-American woman residing in United States was admitted to the hospital with a four-day history of mental status changes complicated by left-sided weakness. Her family reported that she had been acting strange, including exhibiting aggressive behavior towards her niece. On interview, the patient reported that her family members were trying to hurt her. This patient also reported auditory hallucinations, stating, “fire alarms were constantly going off in her house.” Her past medical history was significant for type II diabetes mellitus, hypertension, breast cancer and hypothyroidism. She did not have any past psychiatric or seizure history. Review of systems was only notable for the issues already discussed. The patient's vital signs were stable with a blood pressure of 122/62 mm Hg, respiratory rate of 14 breaths per minute, heart rate of 98 beats per minute and a temperature of 36.4º centigrade. On examination, the patient was awake, alert, and oriented only to her name. Examination of heart, lungs and abdomen were within normal limits. Thyroid examination was likewise within normal limits. Her neurological examination revealed disorientation to place and time, poor memory, agraphia, dyslexia, and an unusual form of aphasia where the patient was observed to be unable to read or write, but was able to spell words on request. Her speech showed lack of fluency and marked hesitation. Her pupils were equal and reactive-to-light. Fundoscopic examination could not be successfully performed due to the presence of bilateral cataracts. Cranial nerve and sensory examination showed no abnormalities. Muscle strength and deep tendon reflexes were within normal limits.

Results of complete blood count testing were within normal limits. Metabolic panel testing was notable for serum glucose of 458 mg/dl. TSH levels were increased to 15 UIU/ml. Microsomal antibodies was raised at 617 IU/ml. Vitamin B12 was normal with value of 258 pg/ml. The patient's RPR was negative. Free T4 was not done due to the patient's refusal. Cerebral spinal fluid analysis was within normal limits. Likewise, the patient's electroencephalogram did not show any seizure activity or evidence of encephalopathy. Her magnetic resonance imaging revealed mild, chronic microangiopathy.

The patient was subsequently given 1000 mg of intravenous Methylprednisolone, repeated daily, for a total of 3 days. This three-day course of high-dose, intravenous steroids resulted in resolution of the patient's symptoms. She was then discharged on an eleven-day course of oral prednisone 60 mg, for a total of fourteen days of therapy. She has subsequently been followed in a community clinic in her home town. To date, no recurrence of symptoms has been reported.

## Discussion

Despite its eponym, Hashimoto's Encephalopathy (HE) [2] is only related to Hashimoto's Thyroiditis by the common presence of anti-thyroid antibodies. Clinical manifestations most often consist of an acute to sub-acute onset of confusion associated with an alteration of consciousness, seizure activity, or myoclonus, and may include hallucinations and delusions.

In reviewing the multitude of conditions that may lead to progressive mental status changes in an elderly patient, such conditions as metabolic derangements, autoimmune conditions, paraneoplastic encephalopathy, infection, psychiatric illness, and progressive neurologic conditions must be considered. HE distinguishes itself through its rapid deterioration in mental status, lack of specific electrolyte or radiographic abnormalities, and specific response to immunosuppressive and immunomodulating agents. Although prion-related encephalopathies, such as Creutzfeldt-Jakob Disease (CJD), are considered in the differential diagnosis of HE, the rapid decline in mental status seen in HE distinguishes it from CJD, which causes a slower decline in functional status, and ultimately results in death [3].

The exact etiology for this neurological state is not known. As elevated immunoglobulin levels are usually seen in this condition, an inflammatory state has been conjectured to be pathologically responsible [4]. Given this condition's similarities to non-vasculitic, autoimmune, inflammatory meningoencephalopathies, it has been suggested that this condition's name should be changed to “Steroid Responsive Encephalopathy Associated with Autoimmune Thyroiditis,” in order to better characterize what we know about the pathophysiology of this condition [5]. Over the last 40 years, the diagnosis of this condition has been hindered by a lack of universally agreed upon diagnostic features. In response to this ambiguity, more clearly defined criteria have been delineated and include encephalopathy with neuropsychiatric features, seizure activity or focal neurologic deficits; presence of antithyroid antibodies; euthyroid status or mild hypothyroidism; no compelling evidence for a more likely etiology, and reversal of symptoms with steroid administration [6].

Laboratory evaluation is critical to making this diagnosis and will typically show an elevated serum level of anti-thyroperoxidase antibody or anti-thyroglobulin antibody. These antibodies, though frequently present, are not felt to be pathogenic, but simply a marker for an underlying disease, possibly an inflammatory meningoencephalopathy. Since cases of HE have been described in patients with a background metabolic milieu in the euthyroid, hyperthroid, and hypothyroid range, HE is felt not be related to thyroid disease. Cerebrospinal fluid analysis (CSF) is abnormal in approximately 80 percent of patients, usually revealing an elevated CSF protein levels. Other CSF findings include lymphocytic pleocytosis as well as the presence of oligoclonal bands and immune complexes [7,8]. In the case of our patient, no such abnormalities were discerned. Nonspecific electroencephalographic (EEG) abnormalities are seen in 90 to 98 percent of patients with the most common finding being nonspecific slowing of background activity [9]. Again, in the case of our index patient, no diagnostic abnormalities were found. Magnetic resonance imaging in patients with Hashimoto's encephalopathy is usually normal. In some patients, particularly those with concomitant cerebral atrophy, T2 signal abnormalities of white matter are noted.

Recognizing that the acute confusional state seen in HE is not related to thyroid status and may exacerbate any mental status changes associated with severe hyperthyroidism or hypothyroidism, correction or near correction of thyroid function abnormalities is frequently required for the diagnosis of this condition. This condition must be considered separate from confusion related to thyroid disease since the addition of immunomodulating agents will be required for its specific treatment. Despite the fact that corticosteroids are widely accepted as the standard treatment for HE, there is no consensus on indications for usage or pharmacologic dosing [10]. There appears to be not consensus on the optimal dose or duration of steroid therapy. One approach advocates a dose of oral Prednisone ranging from 50 to 150 mg each day. Another approach recommends the use of high dose, intravenous Methylprednisone, although there are no studies comparing outcomes using the different administration and dosing strategies. In the case series described by Castillo et al., seventeen of twenty patients received a five-day course of Methylprednisolone 1 gram each day for five days followed by oral steroids in six of these patients. The three remaining patients received between ten to thirty days of oral Prednisone, ranging in doses from 60-100 mg. No difference was mentioned in outcomes, and eight of the total twenty patients (40%) followed, were able to discontinue steroid therapy without a relapse in symptoms [6].

In the case of patients who cannot tolerate corticosteroids or who do not respond to corticosteroids, agents such as Azathioprine and Cyclophosphamide have been employed. In other refractory cases, clinical improvement has been documented using either intravenous immunoglobulin or plasmapheresis [11].

## Conclusion

Hashimoto's encephalopathy is an uncommon complication of thyroid disease, which is readily treatable with conventional modes of pharmacotherapy. As our case and the available literature show, a rapid reversible in the patient's confusion and neurologic debility can be affected after the initiation of therapy. Furthermore, the literature maintains that this disease entity distinguishes itself from confusional states associated with thyroid hormone derangements by its response to steroids or other therapies for autoimmune disease. Our patient exhibited such a response. Thus, although this condition may be considered rare, its sensitivity to medical intervention necessitates that we consider it in our differential diagnosis for acute, and possibly long-term, confusional states; if we do not, we will miss a readily reversible cause for mental status changes.

## List of abbreviations

CSF: Cerebro Spinal Fluid
CT: Computed Tomography
EEG: Electroencephalogram
HE: Hashimoto's Encephalopathy
RPR: Rapid Plasma Reagin
SREAT: Steroid Responsive Autoimmune Thyroiditis
TSH: Thyropid Stimulating Hormone
